# Clinical application of cell-free next-generation sequencing for infectious diseases at a tertiary children’s hospital

**DOI:** 10.1186/s12879-021-06292-4

**Published:** 2021-06-11

**Authors:** Julianne Wilke, Nanda Ramchandar, Christopher Cannavino, Alice Pong, Adriana Tremoulet, Leidy Tovar Padua, Helen Harvey, Jennifer Foley, Lauge Farnaes, Nicole G. Coufal

**Affiliations:** 1 Department of Pediatrics, University of California, 3020 Children’s Way, San Diego, CA 92123 USA; 2 Department of Pediatrics, Rady Children’s Hospital of San Diego, San Diego, USA

**Keywords:** Metagenomics, Cell-free plasma, Next-generation sequencing, Children

## Abstract

**Background:**

Children affected by infectious diseases may not always have a detectable infectious etiology. Diagnostic uncertainty can lead to prolonged hospitalizations, inappropriately broad or extended courses of antibiotics, invasive diagnostic procedures, and difficulty predicting the clinical course and outcome. Cell-free plasma next-generation sequencing (cfNGS) can identify viral, bacterial, and fungal infections by detecting pathogen DNA in peripheral blood. This testing modality offers the ability to test for many organisms at once in a shotgun metagenomic approach with a rapid turnaround time. We sought to compare the results of cfNGS to conventional diagnostic test results and describe the impact of cfNGS on clinical care in a diverse pediatric population at a large academic children’s hospital.

**Methods:**

We performed a retrospective chart review of hospitalized subjects at a tertiary pediatric hospital to determine the diagnostic yield of cfNGS and its impact on clinical care.

**Results:**

We describe the clinical application of results from 142 cfNGS tests in the management of 110 subjects over an 8-month study period. In comparison to conventional testing as a reference standard, cfNGS was found to have a positive percent agreement of 89.6% and negative percent agreement of 52.3%. Furthermore, 32.4% of cfNGS results were directly applied to make a clinical change in management.

**Conclusions:**

We demonstrate the clinically utility of cfNGS in the management of acutely ill children. Future studies, both retrospective and prospective, are needed to clarify the optimal indications for testing.

**Supplementary Information:**

The online version contains supplementary material available at 10.1186/s12879-021-06292-4.

## Introduction

Children affected by infectious diseases may not always have a detectable infectious etiology. Diagnostic uncertainty can lead to prolonged hospitalizations, inappropriately broad or extended courses of antibiotics, invasive diagnostic procedures, and difficulty predicting the clinical course and outcome.

Cell-free plasma next-generation sequencing (cfNGS) can identify viral, bacterial, and fungal infections by detecting pathogen DNA in peripheral blood. This testing modality offers the ability to test for over 1000 organisms at once in a metagenomic approach with a rapid turnaround time of approximately 48 h [1–3]. This technique has successfully identified pathogens in cases with antibiotic pre-exposure and in deep-seated infections that traditionally require invasive sampling of infected tissue [2, 4].

The clinical use of cfNGS has been evaluated in adults with sepsis, oncology patients, and healthy children with community acquired pneumonia [1, 4, 5]. There is a growing body of evidence reporting the clinical application of cfNGS. While some studies demonstrate promising diagnostic applications when compared to conventional diagnostic testing, recent studies have found limited clinical impact when test results are applied directly to patient care [6–9].

In this study, we sought to compare the results of cfNGS to conventional diagnostic test results and describe the impact of cfNGS on clinical care in a diverse pediatric population at a tertiary, academic children’s hospital.

## Methods

### Subject population

This is a single-center, retrospective review of subjects admitted at a tertiary children’s hospital between June 1, 2017 and January 22, 2018 for whom cfNGS was included as part of their diagnostic workup. The Institutional Review Board (IRB) of Rady Children’s Hospital San Diego (RCHSD) and the Human Research Protections Program at the University of California San Diego approved this study. **All methods were performed in accordance with the relevant guidelines and regulations. A waiver of informed consent was granted by the IRB** of Rady Children’s Hospital San Diego and the Human Research Protections Program at the University of California San Diego **for this retrospective study.**

### Cell free next generation sequencing

Peripheral blood samples were collected in a BD Vacutainer Plasma Preparation and sent to the Karius CLIA laboratory (Redwood City, CA) for next-generation sequencing. Sample analysis techniques have been previously described [1]. All subjects from whom cfNGS was obtained had pediatric infectious disease consultation, which guided the decision to obtain cfNGS. Our institution does not have standardized criteria for ordering cfNGS. The decision to obtain cfNGS testing is at the discretion of the consulting infectious disease attending.

### Chart review process

Charts were reviewed to obtain objective subject information. Study data were collected and managed using the Research Electronic Data Capture (REDCap) electronic data capture tools hosted at Rady Children’s Hospital, San Diego, CA. REDCap is a secure, web-based application designed to support data capture for research studies.

### Comparing conventional diagnostic test results

The results of cfNGS were compared to all standard-of-care diagnostic tests obtained within 3 days before or after cfNGS testing in order to better ensure that the comparison of cfNGS to conventional testing reflected a similar clinical picture. All non-cfNGS diagnostic tests sent during this period were considered “conventional diagnostic testing,” and included any diagnostic testing available to our institution including cultures from blood, respiratory aspirate, body fluid, and tissue samples in addition to targeted, pathogen-specific real-time PCR studies (from blood, cerebral spinal fluid, or respiratory swabs).

An organism identified by either conventional diagnostic testing or cfNGS was determined to be “pathologically significant” if the clinical team indicated that the result was plausible, if knowledge of the positive test was employed to make a clinical change in patient management, or retrospectively by an adjudication review of an infectious disease physician independent of the treating physicians. For organisms identified by cfNGS, each organism was separately adjudicated as a true positive (TP), false positive (FP), true negative (TN), or false negative (FN). The cfNGS test was than adjudicated as a single TP, FP, TN, or FN result, reflecting an aggregate assessment of the overall test result in the clinical context of that subject*.* cfNGS as a whole was adjudicated as TP if at least one organism identified by cfNGS was concordant with conventional testing or if at least one organism was deemed sufficient to the clinical scenario for that subject. Conventional testing was adjudicated as an aggregate of all conventional tests sent per subject. A reference standard was then generated from the adjudication of the conventional testing as pathogenic or not by an independent review panel. This application of a reference standard has been previously described [1, 6, 9]. We used this composite reference standard to estimate positive percent agreement (PPA) and negative percent agreement (NPA).

### Clinical application of cfNGS results

Clinical changes to patient management made in response to cfNGS results were assessed by manual chart review. Additionally, a retrospective survey was completed for each cfNGS test by the infectious disease physician who ordered the test to assess the clinical impact. If this physician was not available, then one of five infectious disease faculty at RCHSD completed the survey. The objective chart review and subjective surveys were compared and evaluated by a third infectious disease physician to ensure concordance.

## Results

### Subjects

A total of 110 subjects over 111 individual hospitalizations met inclusion criteria. A total of 142 discrete cfNGS tests were obtained (18 subjects had more than one cfNGS tests sent during their hospitalization).

The most common admission diagnoses fell under infectious, respiratory, or hematological categories. Examples within these categories include sepsis, pneumonia, and febrile neutropenia. There is overlap between diagnostic categories of admission diagnosis. The most common co-morbidities at time of admission include immunocompromised status, pre-existing oncologic diagnoses, and the presence of a pre-existing central line. Over half (53.2%) of the subjects were admitted to an intensive care setting (neonatal, pediatric, or cardiac intensive care units) during their hospitalization (Table 1).

**Table 1:** Demographics

**Subject encounters** ***n*** **= 111**			
	Median age at admission (IQR)	8.2	(2.05–13.5)
	Male	65	58.56%
	Median length of stay days (IQR)	13.9	(8.05–35.9)
**Primary Admission Diagnosis n, %**			
	Infectious	56	50.45%
	Respiratory	39	35.14%
	Hematology/oncology	26	23.42%
	Cardiology	17	15.32%
	Gastroenterology	8	7.21%
	Surgical	6	5.41%
	Nephrology	4	3.60%
	Neurology	1	0.90%
**Comorbidities on Admission n, %**^a^			
	Immunosuppression	37	33.33%
	Oncological diagnosis	25	22.52%
	Central line	24	21.62%
	Cognitive impairment	20	18.02%
	Congenital heart disease	16	14.41%
	Internal hardware	10	9.01%
	Cerebral palsy/motor delay	8	7.21%
	Chronic lung disease	8	7.21%
	Prematurity	8	7.21%
**Admission to Intensive Care Unit n (%)**		59	53.15%
	Pediatric ICU	39	35.13%
	Neonatal ICU	7	6.30%
	Cardiovascular ICU	19	17.12%
**Hospitalization Complications**			
	Respiratory failure	75	67.57%
	Operative intervention	49	44.14%
	Corticosteroids	49	44.14%
	Vasoactive medications	39	35.14%
	Immunoglobulin therapy	20	18.02%
	Death	8	7.21%
	Acute renal failure	7	6.31%
	ECMO	6	5.41%

^a^values add up to more that 100% because comorbidities were not mutually exclusive

### Conventional diagnostic test results

Conventional diagnostic tests obtained during the study period for this cohort of subjects included 224 blood cultures, 142 respiratory pathogen PCR tests, 133 body fluid culture (includes urine and pleural fluid), 97 respiratory cultures from endotracheal tubes or tracheostomies and 402 other tests (Supplemental Table 1). Only 17.1% of the 998 conventional diagnostic tests that were sent returned a positive result, which includes contaminants and false positives.

### cfNGS results

The most common indication for cfNGS testing was clinical symptoms of infection followed by focal imaging findings (Table 2). The median day on which cfNGS was obtained was hospitalization day 6 (IQR 3–6). The median time to cfNGS result availability was 72 h (IQR 48–104). There were 105 tests that identified at least one infectious organism, 59 of which identified more than one organism.

**Table 2:** cfNGS Test Profile

Total cfNGS tests obtained		*n* = 142	%
**Primary Testing Indication**			
	Clinical symptoms suggestive of infection	68	47.9%
	Focal imaging finding	38	26.8%
	Infection monitoring	15	10.6%
	Immunocompromised	9	6.3%
	Validation of conventional testing	6	4.2%
	Elevated inflammatory markers	3	2.1%
	Suspected rheumatologic process	3	2.1%
**Median hospital day sent (IQR)**		6	(3–28.75)
**Hours to results**			
	Mean (SD)	85.7	(38.9)
	Median (IQR)	72	(48–105)
	Mode	48	
**Results**		**n**	**%**
	Positive	105	73.9%
	Single organism	46	
	Multiple organisms	59	
	Negative	37	26.1%

When comparing cfNGS results to conventional testing, we found that of the 105 positive cfNGS tests, 27 (25.7%) identified the same organism as conventional tests. There were 92 tests that identified one or more additional organisms not previously identified by conventional diagnostic testing. This includes the 51 cfNGS tests that identified an organism while all conventional testing was negative (Supplemental Table 2).

Twenty-two cfNGS tests were negative and agreed with negative conventional testing. There were four negative cfNGS tests with no conventional testing available for comparison. There were 11 cfNGS tests that were negative when conventional testing identified an infectious source.

### Interpretation and clinical application

Of the positive cfNGS tests, 69 (65.7%) were considered clinically relevant and were considered true positives. We calculated PPA and NPA data of cfNGS by comparison against the composite reference derived from adjudication of conventional testing as previously described [1, 6]. Conventional diagnostic testing had a PPA of 42% whereas cfNGS had a PPA of 89.6%. The NPA of conventional diagnostic testing was higher than that of cfNGS at 83.9 and 52.3% respectively (Supplemental Table 3).

Chart review determined that 32.4% of cfNGS results were directly applied to make a clinical change in management. This number includes the clinical application of eight tests with negative results. The most common clinical changes were changes to antimicrobial medication regimen: narrowed broad spectrum coverage in 46.7% and shortened duration of treatment in 28.9%. One subject underwent pneumonectomy because of *Rhizopus oryzae* identified on cfNGS (later confirmed by culture of resected lung tissue). The ordering physician reported that the results of the cfNGS was helpful in guiding patient care for 52.1% of the tests sent, which was higher than the percentage change of management (Table 3).

**Table 3:** Clinical Changes and implication

Did the cfNGS result change patient management?			n	%
**Yes**			46	32.4%
	Medication Change		45	
		Narrowed antibiotic coverage	21	
		Broadened antibiotic coverage	7	
		Shortened duration of treatment	13	
		Lengthened duration of treatment	4	
	Procedure performed		1	0.7%
	Effected length of hospitalization		1	0.7%
**No**			92	64.8%
**Unsure**			4	2.8%
**Does the ordering physician believe the cfNGS test was helpful for patient care?**				
		YES	74	52.1%
		NO	63	44.4%
		UNSURE	5	3.5%

## Discussion

This study outlines an institutional experience applying cell-free next generation sequencing from blood samples to the care of hospitalized children over an 8-month study period. We describe the clinical application of results from 142 cfNGS tests in the management of 110 subjects over 111 individual hospitalizations. In comparison to conventional testing as a reference standard, cfNGS determined to have a PPA of 89.6% and NPA of 52.3%. For 92 of the 142 cfNGS tests (64.8%) in which a putative pathogenic organism was determined to be clinically relevant, the relevant organisms were not identified by conventional testing. Furthermore, 32.4% of cfNGS results were directly applied to make a clinical change in management. This study illustrates an increased diagnostic yield for the detection of a putative pathogenic organism in subjects with cfNGS in comparison to conventional testing. Similar to previous studies, cfNGS had a higher rate of positivity for immunocompromised subjects, which is best explained by an increased pre-test probability of infection in these subjects [5, 10–12].

In the acute critical care setting, rapid diagnosis and treatment can improve patient outcomes. cfNGS offers the ability to test for many organisms at once in a broad metagenomic approach with a rapid turnaround time [1, 2, 7]. In a recent study of 350 septic adults, cfNGS demonstrated an increased yield in pathogen detection by approximately 34% over standard of care testing with an average return of result in 29 h [1]. With a rapid turnaround time and a broad range of pathogens detected by this technology, cfNGS may be a useful diagnostic tool in evaluating acutely ill patients, especially where expected diagnostic yield of conventional testing is expected to be low [4, 10, 13]. In our cohort, some cfNGS tests were specifically obtained to trend clearance of an infection or to support an alternative non-infectious diagnosis by obtaining a negative result. We furthermore suspect that the sensitivity and positive predictive value of cfNGS varies based on the clinical context in which testing is sent. For instance, cfNGS tests sent during cardiac admissions for post-operative fevers seem more likely to be negative than in an immunosuppressed subject admitted with sepsis.

Our results demonstrate both a higher degree of clinical utility and positivity of cfNGS than previously reported. In a recent study of the clinical utility of 82 cfNGS tests from mixed adults and pediatric subjects, positivity was found in 50 of 82 samples (61.0%), but with a clinical impact in just 6 (7.3%) instances [9]. This is in contrast to a single center retrospective review of 100 cfNGS results in pediatric subjects, for whom the study authors estimated sensitivity and specificity for diagnosis of a clinically relevant infection was 92 and 64% in a cohort heavily skewed towards oncology patients [6]. There are few studies that have validated cfNGS for specific disease etiologies, and in these studies, understanding results in relation to a historic reference standard as well as what constitutes a commensal organism has been challenging [14, 15]. The real-life interpretation of a test result is therefore ultimately left to the discretion of the treating clinicians, which in the case of this study was always done in collaboration with the infectious disease service.

Interestingly, treating physicians found the test to be useful even if it did not directly change clinical management. Perhaps, confirming standard of care testing may provide a sense of confidence when diagnosis and management decisions remain uncertain. This may provide an unmeasurable type of clinical utility to a provider. Treating a known infection may allow providers a chance to resist the urge to change antimicrobial coverage, especially in severely ill patients with prolonged and complicated infectious courses. In turn, this may mitigate the long-term emergence of resistant organisms.

### Study limitations

This study evaluated cfNGS tests sent in hospitalized children at a single center with limited sample size. Given the retrospective nature of our analysis, the categorization of test indication and determination of clinical relevance of the test are subject to the recall bias of providers. In cases where anti-microbial changes or other changes in management were made, we did not collect data on long term follow up to determine the success of therapy. As is the case with previous studies evaluating cfNGS, the interpretation of the results as a representation of a true infection is also inherently subjective. When cfNGS results with numerous organisms, it may be more difficult to determine an organism’s likelihood of causation of symptoms, especially with organisms that may be considered commensal or otherwise part of normal flora. As cfNGS technology is evolving, however, quantitative values of organism DNA are now available and may help delineate likelihood of pathogenicity. The current available version of this test allows for only detection of organisms through DNA, so RNA based viruses such as SARS-CoV-2 would not be detected using current technology. This limitation could be addressed by also examining RNA. The benefit of hypothesis free testing can be clearly seen when a shortage of specific tests is made apparent. Finally, a standardized approach to guide test indication for both cfNGS and conventional testing does not exist at our institution, allowing for practice variation to potentially confound our results and limit generalizability. Furthermore, given the heterogeneity in the standard care evaluation of these subjects, a systematic comparison of cfNGS to a standardized reference was not possible, further limiting the interpretation and generalizability of our sensitivity analysis. We additionally adjudicated conventional testing as an aggregate of all non-cfNGS tests sent, which, given inherent differences in sensitivity and specificity of each available test, further adds unintended variability in the comparison between cfNGS and conventional testing.

## Conclusion

In a retrospective review of the results of 142 cfNGS tests, we demonstrate the clinically utility in the management of acutely ill children. Future studies, both retrospective and prospective, are needed to clarify the optimal indications for testing, especially in relation to timing of testing within a hospitalization.

## Supplementary Information

**Additional file 1: Supplemental Table 1**. Conventional Test Results. **Supplemental Table 2**. Conventional Testing vs cfNGS Results. **Supplemental Table 3**. PPA and NPA calculations.

**Additional file 2.** Full conventional and cfNGS results with adjudication.

## Data Availability

The datasets generated and/or analyzed during the current study are not publicly available due inclusion of protected health information, but are available from the corresponding author on reasonable request. Sequencing was obtained via the Karius (Redwood City, CA) cfNGS commercial assay, and the sequencing data is therefore not available for public access due to proprietary concerns.

## Abbreviations

cfNGS: Cell-free plasma next-generation sequencing
RCHSD: Rady children’s hospital San Diego
PPA: Positive percent agreement
NPA: Negative percent agreement
