# Supplementary material for: Clinical application of cell-free next-generation sequencing for infectious diseases at a tertiary children’s hospital
Source: BMC Infect Dis. 2021 Jun 11;21:552. doi: 10.1186/s12879-021-06292-4 (PMC8192220; doi:10.1186/s12879-021-06292-4)
Supplement: Supplementary file 1 — Additional file 1: Supplemental Table 1. Conventional Test Results. Supplemental Table 2. Conventional Testing vs cfNGS Results. Supplemental Table 3. PPA and NPA calculations. [file 12879_2021_6292_MOESM1_ESM.docx]

**Supplemental Table 1: Conventional Test Results**

|  | **Total tests** | **Number positive tests** | | **Number negative tests** | |
| --- | --- | --- | --- | --- | --- |
| Other | 402 | 54 | 13.4% | 348 | 86.6% |
| Blood culture | 224 | 12 | 5.4% | 212 | 94.6% |
| Respiratory Pathogen PCR | 142 | 35 | 24.6% | 107 | 75.4% |
| Body fluid culture | 133 | 33 | 24.8% | 100 | 75.2% |
| Respiratory culture | 68 | 36 | 52.9% | 32 | 47.1% |
| Fungal culture | 29 | 1 | 3.4% | 28 | 96.6% |

| **Supplemental Table 2: Conventional Testing vs cfNGS Results** | | |  |  |
| --- | --- | --- | --- | --- |
| **cfNGS Results** | | | n (%) |  |
|  | **cfNGS positive** | | n = 105 |  |
|  |  | cfNGS results same as conventional | 27 (25.7) |  |
|  |  | cfNGS identified organism not found by conventional testing | 92 (87.6) |  |
|  |  | Conventional negative | 51 (48.6) |  |
|  |  | Conventional found something new | 37 (35.2) |  |
|  | **cfNGS Negative** | | N = 37 |  |
|  |  | cfNGS and Conventional tests negative | 22 (59.5) |  |
|  |  | cfNGS Negative and Conventional positive | 11 (29.7) |  |
|  |  | cfNGS Negative and no Conventional test sent | 4 (10.8) |  |

**Supplemental Table 3: PPA and NPA calculations**

| Conventional Testing |  |  |  | cfNGS Testing |  |  |
| --- | --- | --- | --- | --- | --- | --- |
|  | Has Disease | No Disease |  |  | Has Disease | No Disease |
| Test Pos | 34 | 9 |  | Test Pos | 69 | 31 |
| Test Neg | 47 | 47 |  | Test Neg | 8 | 34 |
|  |  |  |  |  |  |  |
| PPA | 0.420 |  |  | PPA | 0.896 |  |
| NPA | 0.839 |  |  | NPA | 0.523 |  |
|  |  |  |  |  |  |  |
| True Positive | 34 |  |  | True Positive | 69 |  |
| False Positive | 9 |  |  | False Positive | 31 |  |
| True Negative | 47 |  |  | True Negative | 34 |  |
| False Negative | 47 |  |  | False Negative | 8 |  |
